# Factors associated with in-hospital heart failure after emergency percutaneous coronary intervention in patients with type 2 diabetes mellitus complicated by ST-segment elevation myocardial infarction: a retrospective study

**DOI:** 10.3389/fendo.2026.1913869

**Published:** 2026-09-03

**Authors:** Mailidan Maimaitiniyazi, Muyesaier Maimaitiniyazi, Meiheliya Maisuti, Tuersunayi Yisimitila, Palida Yushanjiang, Muyesai Nijiati

**Affiliations:** 1 Emergency Center, People’s Hospital of Xinjiang Uygur Autonomous Region, Urumqi, China; 2 Xinjiang Medical University, Urumqi, Xinjiang, China

**Keywords:** heart failure, percutaneous coronary intervention, risk factors, ST-segment elevation myocardial infarction, type 2 diabetes mellitus

## Abstract

**Background:**

Type 2 diabetes mellitus (T2DM) is associated with adverse outcomes in patients with ST-segment elevation myocardial infarction (STEMI). Despite emergency percutaneous coronary intervention (PCI), in-hospital heart failure (HF) remains an important complication. This study aimed to identify factors independently associated with new-onset in-hospital HF and to develop an internally validated prediction model in patients with T2DM and STEMI undergoing emergency PCI.

**Methods:**

This retrospective observational study included 306 adult patients with T2DM and STEMI who underwent emergency PCI at the People’s Hospital of Xinjiang Uygur Autonomous Region between January 2022 and February 2026. Patients were classified into HF (n=65) and non-HF (n=241) groups according to new-onset clinically documented HF during the index hospitalization; all patients classified as having HF had a maximum Killip class ≥ II. Natriuretic peptides and echocardiographic findings were used as supportive evidence and were not included as predictors. Clinical, laboratory, echocardiographic, and selected procedural variables were analyzed using univariate and multivariable binary logistic regression. Model performance was assessed using discrimination, calibration, 10-fold cross-validation, and bootstrap optimism correction.

**Results:**

Independent positive associations with in-hospital HF were observed for prior myocardial infarction (OR = 6.205, 95% CI: 2.692–14.299, P<0.001), smoking (OR = 5.552, 95% CI: 2.517–12.247, P<0.001), regional wall motion abnormalities (OR = 2.415, 95% CI: 1.091–5.344, P = 0.030), higher white blood cell count (OR = 1.143 per 10^9^/L, 95% CI: 1.072–1.219, P<0.001), higher systolic blood pressure (OR = 1.018 per mmHg, 95% CI: 1.001–1.034, P = 0.032), and age ≥75 years (OR = 4.792, 95% CI: 1.394–16.472, P = 0.013). Hemoglobin (OR = 0.971 per 1 g/L increase, 95% CI: 0.952–0.990, P = 0.003) and LVEF (OR = 0.953 per 1 percentage-point increase, 95% CI: 0.911–0.998, P = 0.040) were inversely associated with HF, indicating greater HF odds at lower values. The model showed an apparent AUC of 0.881 and an optimism-corrected AUC of 0.843, with Hosmer–Lemeshow P = 0.826.

**Conclusion:**

Readily available admission variables were associated with in-hospital HF in T2DM-STEMI patients undergoing emergency PCI. The internally validated model showed good discrimination and calibration; however, given the retrospective single-center design and limited number of HF events, external validation in an independent population is required before clinical implementation.

## 1 Introduction

Heart failure (HF) remains a leading cause of morbidity and mortality worldwide and constitutes a major public health burden. Despite substantial progress in guideline-directed pharmacological and interventional therapies, the prognosis of HF remains poor, with an estimated 5-year mortality approaching 50% (1). Therefore, improving early prevention and risk stratification strategies for HF remains a critical priority in cardiovascular research and clinical practice.

Acute myocardial infarction (AMI) is one of the leading precipitants of HF, primarily through extensive myocardial necrosis and subsequent adverse ventricular remodeling (2). Among patients presenting with ST-segment elevation myocardial infarction (STEMI), HF frequently develops during hospitalization or early follow-up, particularly in those with large infarct size or delayed reperfusion. Despite timely reperfusion with primary percutaneous coronary intervention (PCI), approximately one-quarter of patients are reported to develop HF during follow-up (3, 4), highlighting the need for improved identification of high-risk subgroups.

Type 2 diabetes mellitus (T2DM) is highly prevalent among patients with STEMI and is consistently associated with worse cardiovascular outcomes. Chronic hyperglycemia and insulin resistance are associated with endothelial dysfunction, systemic inflammation, oxidative stress, and microvascular impairment, thereby accelerating myocardial injury and maladaptive remodeling (5, 6). In the era of widespread PCI, emergency PCI has significantly improved short-term survival in STEMI. Nevertheless, patients with concomitant T2DM remain particularly vulnerable to developing in-hospital HF even after successful reperfusion therapy (7, 8). However, evidence regarding early predictors of in-hospital HF after PCI in this high-risk population remains limited, and clinically practical predictive indicators are still needed.

In light of these considerations, this study aimed to identify factors independently associated with in-hospital HF among patients with T2DM presenting with STEMI and treated with emergency PCI. We further developed and internally validated a multivariable prediction model to evaluate its potential for early risk stratification in this high-risk population.

## 2 Methods

### 2.1 Study design and setting

This retrospective observational study was conducted at the People’s Hospital of Xinjiang Uygur Autonomous Region between January 1, 2022, and February 1, 2026.

### 2.2 Study population

**Figure 1:**
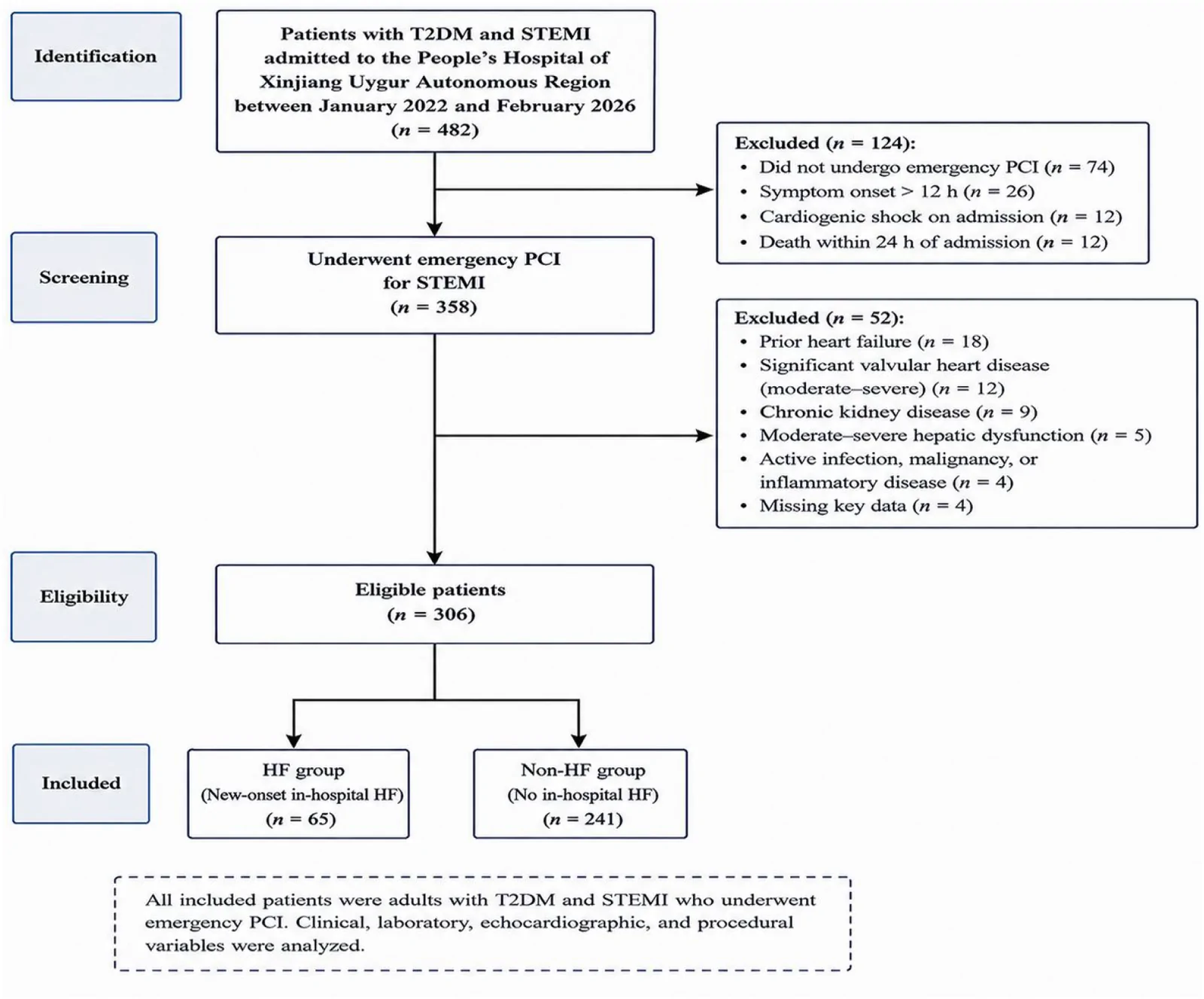
Flowchart of patient screening and study cohort selection.

A total of 306 adult patients (≥18 years) with T2DM and STEMI who underwent emergency PCI were included in the final analytic cohort. Patients were categorized according to the occurrence of new-onset in-hospital HF into an HF group (n = 65) and a non-HF group (n = 241). The cohort comprised 209 males and 97 females, with an age range of 50–95 years and a mean age of 62.497 ± 9.187 years. The patient screening and study cohort selection process is shown in **Figure 1**.

#### 2.2.1 Inclusion criteria

(1) Age ≥ 18 years.
(2) Diagnosis of T2DM according to established criteria (9), meeting any one of the following: (1) Fasting plasma glucose ≥ 7.0 mmol/L; (2) 2-hour plasma glucose ≥ 11.1 mmol/L during an oral glucose tolerance test; (3) Glycated hemoglobin (HbA1c) ≥ 6.5%; (4) Random plasma glucose ≥ 11.1 mmol/L in the presence of classic symptoms of hyperglycemia; or a previously established diagnosis of diabetes mellitus with ongoing glucose-lowering therapy.
(3) Diagnosis of acute STEMI based on established criteria (10), including ischemic chest pain, new or presumed new significant electrocardiographic changes in at least two contiguous leads (ST-segment elevation and/or T-wave changes), and abnormal cardiac biomarkers (a rise and/or fall in cardiac troponin values with at least one value above the 99th percentile upper reference limit), further confirmed by coronary angiography and transthoracic echocardiography.

The primary outcome was new-onset, clinically documented in-hospital HF occurring after emergency PCI during the index hospitalization. HF was identified on the basis of compatible new-onset clinical symptoms and/or signs of HF (11), such as dyspnea, pulmonary rales, jugular venous distension, pulmonary edema, or peripheral edema, together with clinical evidence consistent with cardiac dysfunction or congestion. In patients classified as having in-hospital HF, the maximum Killip class during hospitalization was ≥ II. Elevated BNP or NT-proBNP levels and echocardiographic evidence of cardiac dysfunction were used as supportive objective evidence when available, rather than as mandatory stand-alone diagnostic criteria. Patients who remained Killip class I without clinically documented HF throughout hospitalization were classified as the non-HF group.BNP, NT-proBNP, and Killip classification were used only for outcome ascertainment and were excluded from predictor modeling.

#### 2.2.2 Exclusion criteria

(1) patients who did not undergo emergency percutaneous coronary intervention (PCI) or had a symptom-to-admission time >12 h; (2) patients presenting with cardiogenic shock on admission or who died within 24 h of admission; (3) patients with pre-existing HF at admission or a prior diagnosis of HF; (4) patients with moderate-to-severe valvular heart disease, cardiomyopathy, or other significant structural heart diseases; (5) patients with chronic kidney disease or moderate-to-severe hepatic dysfunction; (6) patients with severe neurological disorders or psychiatric illnesses; (7) patients receiving medications known to significantly affect cardiac function; (8) patients with concomitant interstitial lung disease, pulmonary embolism, or other conditions that may interfere with the diagnosis of HF; (9) patients with active infection, malignancy, systemic inflammatory disease, hematologic diseases, hemolytic anemia, aplastic anemia, or other primary blood disorders that may independently cause abnormalities in hemoglobin or white blood cell counts; and (10) patients with missing key clinical, laboratory, echocardiographic, or procedural data required for the analysis.

The study protocol was approved by the Medical Ethics Committee of the People’s Hospital of Xinjiang Uygur Autonomous Region. This study was conducted in accordance with the Declaration of Helsinki and all methods involving human participants were performed in accordance with relevant guidelines and regulations.

Clarification regarding chronic kidney disease: the exclusion criterion referred to a documented pre-existing diagnosis of chronic kidney disease. An isolated low eGFR measured at admission was retained as a renal-function finding and was not, by itself, considered evidence of established chronic kidney disease.

### 2.3 Clinical data collection

Baseline predictor variables were measured at admission, before emergency PCI, and before the occurrence of in-hospital HF whenever temporally applicable. Demographic characteristics, medical history, vital signs, baseline laboratory measurements, echocardiographic parameters, and selected PCI-related variables were obtained through review of the electronic medical records.

Recorded variables included age, sex, hypertension, prior myocardial infarction, hyperlipidemia, chronic obstructive pulmonary disease (COPD), smoking, systolic and diastolic blood pressure, body mass index (BMI), atrial fibrillation, hemoglobin, WBC count, platelet count, albumin, globulin, serum creatinine, blood urea nitrogen, serum potassium, total cholesterol, LVEF, LVESD, LVEDD, aortic root diameter, regional wall motion abnormalities;Coronary angiographic and procedural characteristics were obtained from the original coronary angiography and PCI procedural records. These variables included the presence of multivessel coronary artery disease, culprit vessel, pre- and post-procedural TIMI flow grades, number of implanted stents, completeness of revascularization, time from chest pain onset to balloon inflation, and door-to-balloon (D-to-B) time.

In-hospital course variables included mechanical ventilatory support, CCU length of stay, and total hospital length of stay. The use of GLP-1 receptor agonists and SGLT2 inhibitors was defined as pre-admission chronic medication use according to medication records obtained before hospitalization. Only baseline exposure status was considered in the analysis, while medications newly initiated during hospitalization were not included.

Multivessel coronary artery disease was defined as significant stenosis involving two or more major epicardial coronary arteries. The culprit vessel was defined as the coronary artery responsible for the acute STEMI event based on angiographic findings. TIMI flow grade was assessed immediately before and after PCI. Complete revascularization was defined as successful treatment of all angiographically significant lesions considered suitable for revascularization during the index hospitalization.

Major in-hospital cardiovascular complications were retrospectively collected during the index hospitalization after emergency PCI. These complications included cardiac death, cardiogenic shock, malignant arrhythmias, requirement for mechanical circulatory support, and recurrent acute myocardial infarction.

### 2.4 Statistical analysis

Descriptive statistics. Continuous variables were expressed as mean ± standard deviation (SD) for normally distributed data or as median with interquartile range (IQR) for non-normally distributed data. Between-group comparisons were performed using Welch’s t-test for continuous variables and the Mann–Whitney U test for skewed distributions (NT-proBNP, BNP). Categorical variables were presented as frequencies and percentages and compared using the Pearson χ² test, or Fisher’s exact test when expected cell counts were < 5. The estimated glomerular filtration rate (eGFR) was calculated using the CKD-EPI 2009 equation without the race coefficient. The missing data rate for each variable ranged from 1.2% to 3.5%, which was low. Missing data were imputed using multiple imputation by chained equations, generating five imputed datasets, and pooled estimates were reported according to Rubin’s rules.

The primary outcome was new-onset, clinically documented in-hospital HF occurring during the index hospitalization after emergency PCI, as defined in Section 2.2. All patients classified as having HF had a maximum Killip class ≥ II, whereas patients who remained Killip class I without clinically documented HF served as the non-HF group. Killip classification, BNP, and NT-proBNP were used only for outcome ascertainment or supportive assessment and were excluded from all predictor models to avoid incorporation bias.

Logistic regression modeling. Univariate binary logistic regression was initially performed for all eligible candidate variables. The multivariable model was constructed using a pre-specified entry approach, incorporating: (i) variables with a univariate p-value < 0.05; (ii) posterior wall myocardial infarction (univariate p = 0.050), retained due to clinical relevance; and (iii) clinically mandated covariates (age, sex, hypertension, and door-to-balloon time) forced into the model. Backward elimination was not employed to preserve clinical interpretability. Results are reported as odds ratios (ORs) with 95% Wald confidence intervals (CIs).

Linearity and variable specification. The linearity of the logit for continuous predictors was assessed using the Box–Tidwell test, which examined the interaction between each continuous variable and its natural logarithm within the full multivariable model. All variables satisfied the linearity assumption except age (p = 0.0055), which was therefore re-specified. Three alternative approaches were compared: (B0) age as a linear term; (B1) age modeled with restricted cubic splines (RCS, with 4 knots placed at the 5th, 35th, 65th, and 95th percentiles per Harrell’s recommendations); and (B2) age categorized at clinically meaningful thresholds (< 60, 60–74, ≥ 75 years). Based on model fit (AIC, BIC), 10-fold cross-validated AUC, and interpretability, model B2 was selected as the primary specification. The RCS-based model (B1) is reported as a sensitivity analysis (**Supplementary Table 2**).

Model performance and diagnostics. Multicollinearity was assessed using variance inflation factors (VIF), with a pre-defined threshold of < 5. Model discrimination was evaluated using the area under the receiver operating characteristic curve (AUC), with three complementary estimates: the apparent AUC, a bootstrap 95% CI (1, 000 replicates), a 10-fold stratified cross-validated AUC, and Harrell’s optimism-corrected AUC (1, 000 bootstrap replicates). Calibration was assessed via the Hosmer–Lemeshow goodness-of-fit test, the calibration intercept (ideal: 0) and slope (ideal: 1.0) from cross-validated predictions, and a graphical calibration plot. Overall model fit was reported using Cox–Snell and Nagelkerke pseudo-R², AIC, BIC, log-likelihood, and the likelihood-ratio test against the null model. The Brier score was also reported as a combined measure of discrimination and calibration.

Clinical utility was evaluated using Decision curve analysis (DCA), computing the net benefit of the model across threshold probabilities ranging from 1% to 80%. This was compared against two reference strategies: “treat all” and “treat none.”Two sensitivity analyses were performed to test the robustness of our findings: (i) Model A, identical to the primary model but excluding posterior wall MI; (ii) Model B1, replacing categorical age with RCS.

Multiple comparisons and model complexity. Given that candidate predictors were tested univariately, a Benjamini–Hochberg false-discovery-rate (FDR) correction was applied as a robustness check. The primary variable-selection rule remained univariate P < 0.05 with forced inclusion of pre-specified clinical covariates. After FDR correction, five variables remained significant at q < 0.05 (**Supplementary Table 3**).

No *a priori* sample-size calculation was performed because this was a retrospective analysis of the available final cohort. The primary model estimated 15 regression coefficients (excluding the intercept; age contributed two indicator terms) with 65 HF events, corresponding to approximately 4.3 events per estimated coefficient. This limited event-to-parameter ratio indicates a meaningful risk of model overfitting. We therefore regard the model as exploratory; 10-fold cross-validation and bootstrap optimism correction were used to quantify internal optimism, but they do not substitute for external validation.

All statistical analyses were performed using Python 3.12 with the following packages: statsmodels 0.14 (for Logit and GLM), scikit-learn 1.x (for StratifiedKFold, cross-validation, and ROC), SciPy 1.x (for statistical tests and bootstrap), and Matplotlib (for figures). All tests were two-sided, and a p-value < 0.05 was considered statistically significant.

## 3 Results

### 3.1 Comparison of baseline characteristics and univariate analysis between the two groups

All variables were compared between the HF group (n = 65) and non-HF group (n = 241). Continuous variables are presented as mean ± SD or median (IQR), and categorical variables as n (%)(**Table 1**). Variables considered for regression are detailed in **Table 2**.

**Table 1:** Comparison of clinical characteristics between HF group and non-HF group.

Variables	HF Group (n = 65)	Non-HF Group (n = 241)	Statistic	p value
Demographic characteristics				
Age (years)	63.723 ± 12.817	62.166 ± 7.933	t = +0.932	0.354
Sex			χ² = 0.014	0.905
Male	44 (67.7%)	165 (68.5%)		
Female	21 (32.3%)	76 (31.5%)		
Medical history				
Hypertension	27 (41.5%)	76 (31.5%)	χ² = 2.294	0.130
★ Prior myocardial infarction	28 (43.1%)	30 (12.4%)	χ² = 31.263	< 0.001 ***
COPD	17 (26.2%)	79 (32.8%)	χ² = 1.044	0.307
Hyperlipidemia	17 (26.2%)	79 (32.8%)	χ² = 1.044	0.307
★ Smoking	45 (69.2%)	90 (37.3%)	χ² = 21.112	< 0.001 ***
Vital signs & anthropometrics				
★ Systolic BP (mmHg)	130.215 ± 22.772	124.004 ± 22.121	t = +1.963	0.052 #
Diastolic BP (mmHg)	78.092 ± 16.738	80.008 ± 17.690	t = -0.809	0.420
BMI (kg/m²)	25.901 ± 3.834	26.361 ± 3.809	t = -0.860	0.392
Echocardiographic parameters				
★ LVEF (%)	48.754 ± 8.553	51.085 ± 7.500	t = -2.000	0.048 *
LVESD (mm)	33.015 ± 8.817	32.614 ± 5.593	t = +0.349	0.728
LVEDD (mm)	49.200 ± 7.218	50.207 ± 6.058	t = -1.032	0.305
Aortic root diameter (mm)	32.200 ± 2.933	31.656 ± 3.631	t = +1.259	0.210
★ Regional wall motion abnormalities	49 (75.4%)	128 (53.1%)	χ² = 10.414	0.001 **
Laboratory parameters				
★ Hemoglobin (g/L)	132.415 ± 21.473	138.842 ± 19.607	t = -2.180	0.032 *
★ WBC count (×10^9^/L)	14.474 ± 6.213	10.083 ± 4.616	t = +5.316	< 0.001 ***
Platelet count (×10^9^/L)	253.200 ± 86.836	232.539 ± 74.129	t = +1.754	0.083 #
Albumin (g/L)	38.920 ± 4.123	39.979 ± 5.627	t = -1.689	0.093 #
Globulin (g/L)	28.956 ± 6.049	27.615 ± 5.545	t = +1.614	0.110
Total cholesterol (mmol/L)	4.285 ± 1.068	4.204 ± 1.261	t = +0.519	0.605
Serum creatinine (μmol/L)	83.232 ± 20.291	78.497 ± 52.713	t = +1.120	0.264
Blood urea nitrogen (mmol/L)	6.661 ± 2.288	6.275 ± 3.709	t = +1.041	0.300
Serum potassium (mmol/L)	4.090 ± 0.512	3.989 ± 0.453	t = +1.458	0.148
HbA1c (%)	7.840 ± 2.396	7.339 ± 2.073	t = +1.537	0.128
★ eGFR (mL/min/1.73 m²)	79.131 ± 19.928	87.854 ± 22.140	t = -3.057	0.003 **
Normal (≥ 90)	20 (30.8%)	139 (57.7%)	χ² = 15.335	< 0.001 ***
Mild impairment (60–89)	34 (52.3%)	72 (29.9%)		
Moderate–severe impairment (< 60)	11 (16.9%)	30 (12.4%)		
Cardiac biomarkers				
NT-proBNP (pg/mL) +	6140 (3800–7240)	265 (190–385)	Z = -12.373	< 0.001 ***
BNP (pg/mL) +	2680 (1650–3150)	85 (74–100)	Z = -12.374	< 0.001 ***
Cardiac rhythm				
★ Atrial fibrillation	13 (20.0%)	24 (10.0%)	χ² = 4.856	0.028 *
Coronary artery lesion characteristics				
Multivessel coronary artery disease	22 (33.8%)	90 (37.3%)	χ² = 0.270	0.603
Anterior wall MI	24 (36.9%)	82 (34.0%)	χ² = 0.190	0.663
Inferior wall MI	30 (46.2%)	91 (37.8%)	χ² = 1.509	0.219
Posterior wall MI	15 (23.1%)	87 (36.1%)	χ² = 3.907	0.048 *
Procedural variables				
Time from chest pain to balloon (h)	6.040 ± 1.611	5.552 ± 1.888	t = +2.085	0.039 *
D-to-B time (min)	81.323 ± 6.086	80.365 ± 3.201	t = +1.224	0.225
In-hospital course				
CCU length of stay (days)	1.908 ± 0.723	1.975 ± 0.718	t = -0.668	0.506
Total hospital length of stay (days)	7.923 ± 1.229	7.934 ± 1.199	t = -0.062	0.951
Mechanical ventilatory support	27 (41.5%)	93 (38.6%)	χ² = 0.187	0.666
Medications				
GLP-1 RA use	9 (13.8%)	33 (13.7%)	χ² = 0.001	0.975
SGLT-2i use	12 (18.5%)	46 (19.1%)	χ² = 0.013	0.909
Socioeconomic factors				
Occupation (employed vs. farmer)	31 (47.7%)	140 (58.1%)	χ² = 2.245	0.134
Medical insurance	46 (70.8%)	170 (70.5%)	χ² = 0.001	0.971

HF, heart failure; BP, blood pressure; BMI, body mass index; LVEF, left ventricular ejection fraction; LVESD, left ventricular end-systolic diameter; LVEDD, left ventricular end-diastolic diameter; WBC, white blood cell; HbA1c, glycated hemoglobin; eGFR, estimated glomerular filtration rate (CKD-EPIformula); NT-proBNP, N-terminal pro-B-type natriuretic peptide; BNP, B-type natriuretic peptide; D-to-B, door-to-balloon; CCU, coronary care unit; GLP-1 RA, glucagon-like peptide-1 receptor agonist; SGLT-2i, sodium-glucose cotransporter-2 inhibitor.

*** p < 0.001; ** p < 0.01; * p < 0.05; # p = 0.05–0.10. ★ Variables with univariate P < 0.05 were considered for multivariable modeling. + Outcome components were excluded.

**Table 2:** Univariate binary logistic regression — all candidate variables.

Variable	OR	95% CI	p value
WBC count (×10^9^/L) ★	1.157	1.098–1.219	< 0.001 ***
Prior myocardial infarction ★	5.323	2.857–9.917	< 0.001 ***
Smoking ★	3.775	2.097–6.795	< 0.001 ***
Regional wall motion abnormalities ★	2.704	1.457–5.018	0.002 **
eGFR (mL/min/1.73 m²) ★	0.983	0.971–0.995	0.005 **
Hemoglobin (g/L) ★	0.985	0.971–0.998	0.024 *
Atrial fibrillation ★	2.260	1.079–4.736	0.031 *
LVEF (%) ★	0.964	0.931–0.997	0.034 *
Systolic BP (mmHg) ★	1.012	1.000–1.024	0.048 *
Posterior wall MI #	0.531	0.282–1.001	0.050 #
Platelet count (×10^9^/L) #	1.003	1.000–1.007	0.059 #
Time from chest pain to balloon (h) #	1.154	0.994–1.340	0.059 #
Globulin (g/L) #	1.045	0.993–1.099	0.092 #
D-to-B time (min) #	1.061	0.990–1.136	0.092 #
HbA1c (%) #	1.105	0.981–1.245	0.099 #
Serum potassium (mmol/L)	1.610	0.885–2.931	0.119
Hypertension	1.543	0.878–2.709	0.131
Occupation (employed vs. farmer)	0.658	0.380–1.140	0.135
Albumin (g/L)	0.966	0.920–1.014	0.161
Inferior wall MI	1.413	0.813–2.456	0.220
Age (years)	1.019	0.989–1.049	0.226
LVEDD (mm)	0.974	0.930–1.019	0.255
Aortic root diameter (mm)	1.046	0.967–1.131	0.266
Hyperlipidemia	0.726	0.393–1.343	0.308
COPD	0.726	0.393–1.343	0.308
BMI (kg/m²)	0.968	0.899–1.042	0.388
Diastolic BP (mmHg)	0.994	0.978–1.009	0.433
Blood urea nitrogen (mmol/L)	1.029	0.958–1.104	0.434
Serum creatinine (μmol/L)	1.002	0.997–1.007	0.493
Multivessel coronary artery disease	0.858	0.482–1.527	0.604
Total cholesterol (mmol/L)	1.055	0.844–1.319	0.636
LVESD (mm)	1.010	0.968–1.053	0.653
Anterior wall MI	1.135	0.642–2.007	0.663
Mechanical ventilatory support	1.131	0.648–1.974	0.666
Sex (male)	0.965	0.537–1.735	0.905
SGLT-2i use	0.960	0.475–1.941	0.909
Medical insurance	1.011	0.554–1.846	0.971
GLP-1 RA use	1.013	0.458–2.241	0.975
Variables excluded from regression analysis +			
NT-proBNP (pg/mL) — outcome component	—	—	—
BNP (pg/mL) — outcome component	—	—	—
CCU length of stay (days) — post-event variable	—	—	—
Total length of stay (days) — post-event variable	—	—	—

in-hospital HF. n = 306 (HF: 65; non-HF: 241). Variables listed in ascending order of p value. Rows marked ★ (p < 0.05) entered the multivariable model; rows marked # had 0.05 ≤ p < 0.10. + Variables not eligible for regression (outcome components or post-event reverse-causality variables) are listed at the bottom.

OR, odds ratio; CI, confidence interval. *** p < 0.001; ** p < 0.01; * p < 0.05; # p = 0.05–0.10.

### 3.2 Multivariable binary logistic regression — final model

Variables with univariate P < 0.05 were entered into the multivariable model, together with posterior wall MI (univariate P = 0.050) and forced-entry covariates (age, sex, hypertension, and D-to-B time). Outcome components were excluded. Age was modeled categorically because the Box–Tidwell test indicated non-linearity. The final model contained 15 estimated coefficients for 65 HF events; therefore, the effect estimates and model performance should be interpreted cautiously in view of possible overfitting (**Table 3**).

**Table 3:** Multivariable binary logistic regression — final model.

Variable	β	SE	OR	95% CI	z	p value
Prior myocardial infarction ▲	+1.825	0.426	6.205	2.692–14.299	+4.285	< 0.001 ***
Smoking ▲	+1.714	0.404	5.552	2.517–12.247	+4.247	< 0.001 ***
Regional wall motion abnormalities ▲	+0.882	0.405	2.415	1.091–5.344	+2.175	0.030 *
WBC count (×10^9^/L) ▲	+0.134	0.033	1.143	1.072–1.219	+4.093	< 0.001 ***
eGFR (mL/min/1.73 m²)	-0.006	0.010	0.994	0.974–1.014	-0.576	0.564
Hemoglobin (g/L) ▲	-0.029	0.010	0.971	0.952–0.990	-2.926	0.003 **
Atrial fibrillation	+0.574	0.540	1.775	0.615–5.120	+1.062	0.288
LVEF (%) ▲	-0.048	0.023	0.953	0.911–0.998	-2.052	0.040 *
Systolic BP (mmHg) ▲	+0.018	0.008	1.018	1.001–1.034	+2.143	0.032 *
Posterior wall MI ▲	-1.056	0.441	0.348	0.147–0.826	-2.395	0.017 *
Age (categorical)						
Age 60–74 (vs. < 60, ref.)	-0.169	0.401	0.844	0.384–1.854	-0.422	0.673
Age ≥ 75 (vs. < 60, ref.) ▲	+1.567	0.630	4.792	1.394–16.472	+2.488	0.013 *
Forced-entry clinical covariates						
Sex (male)	+0.193	0.443	1.213	0.509–2.892	+0.435	0.663
Hypertension	+0.285	0.388	1.330	0.622–2.845	+0.735	0.463
D-to-B time (min)	+0.025	0.044	1.025	0.940–1.118	+0.568	0.570

β, regression coefficient; SE, standard error; OR, odds ratio (exp[β]); CI, confidence interval; z, Wald statistic. *** p < 0.001; ** p < 0.01; * p < 0.05. Age categories: < 60 (n = 123) reference; 60–74 (n = 149); ≥ 75 (n = 34).

For continuous predictors, ORs are expressed per unit increase. The negative coefficients for hemoglobin (β = -0.029) and LVEF (β = -0.048) correspond to ORs <1; thus higher values are inversely associated with HF odds, whereas lower values are associated with higher HF odds. No reverse coding of these variables was used.

### 3.3 Model performance and diagnostics

**Figure 2:**
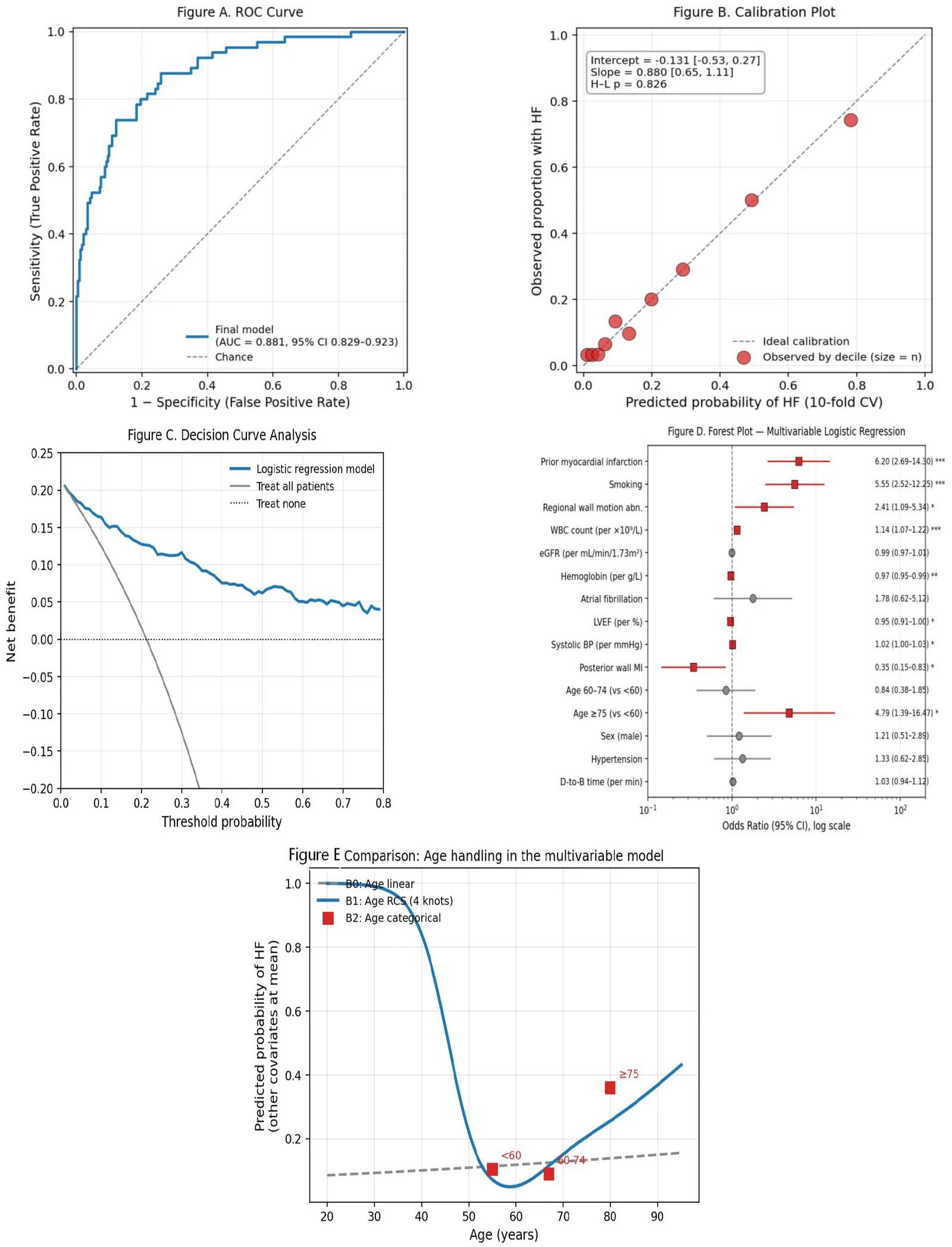
**(A)** ROC Curve. **(B)** Calibration Plot. **(C)** Decision Curve Analysis. **(D)** Forest Plot–Multivariable Logistic Regression. **(E)** Comparison: Age handling in the multivariable. To illustrate the rationale for categorical age modelling, **(E)** compared the three age-specification approaches (linear, restricted cubic splines, and categorical). The linear specification (B0) failed to capture the elevated HF risk after 75 years and the relatively attenuated risk in the 60–74 years subgroup; the categorical specification (B2, < 60, 60–74, ≥ 75 years) effectively captured this non-linear pattern while providing clinically interpretable risk groups, supporting its selection for the primary analysis.

The final multivariable logistic model achieved an apparent AUC of 0.881 (95% bootstrap CI: 0.829–0.924). The 10-fold cross-validated AUC was 0.838, and Harrell’s optimism-corrected AUC was 0.843, with a mean optimism of 0.038. Calibration was assessed by the Hosmer–Lemeshow test (P = 0.826), a calibration intercept of -0.13 (95% CI: -0.53 to 0.27), and a calibration slope of 0.88 (95% CI: 0.65–1.11). These results indicate good internal performance, but the model has not been externally validated. All variance inflation factors were <1.5 (**Table 4**, **Figures 2A–E**).

**Table 4:** Model performance and diagnostics.

Metric	Value
Sample and model	
Total sample size	306
Events (in-hospital HF)	65
Log-likelihood	-100.737
Likelihood-ratio test vs. null (p)	< 0.001
AIC	233.47
BIC	293.05
Discrimination	
Apparent AUC	0.8813
Apparent AUC, 95% bootstrap CI (1, 000 replicates)	0.8288–0.9235
10-fold cross-validated AUC	0.8378
Optimism-corrected AUC (Harrell, 1, 000 bootstraps)	0.8432
Mean optimism	0.0381
Calibration	
Hosmer–Lemeshow χ² (10 deciles)	4.330 (df = 8)
Hosmer–Lemeshow p	0.826
Calibration intercept (ideal = 0), 10-fold CV	-0.131 [95% CI -0.53, 0.27]
Calibration slope (ideal = 1), 10-fold CV	0.880 [95% CI 0.65, 1.11]
Overall fit and accuracy	
Cox–Snell pseudo-R²	0.3133
Nagelkerke pseudo-R²	0.4861
Brier score	0.1026
Multicollinearity	
Maximum VIF (all model variables)	1.366
Number of variables with VIF ≥ 5	0

### 3.4 Supplementary and sensitivity analyses

Several supplementary analyses were performed to assess model assumptions, variablespecification, multiplicity control, and robustness of the findings (**Supplementary Tables 1**–**4**).

#### 3.4.1 Linearity assumption

The Box–Tidwell test was applied to all continuous predictors in the full multivariablemodel by introducing an X × ln(X) interaction term for each variable. As shown in **Supplementary Table 1**, all continuous predictors satisfied the linearity assumption (p>0.05) except age (p=0.005), which was therefore re-specified as a categorical variable in the primary model.

#### 3.4.2 Comparison of age modelling strategies

Comparison of three specifications of age in the same multivariable model (all other covariates identical). Model B2 (categorical) was selected as the primary specification on the basis of clinical interpretability and competitive fit; B1 (restricted cubic spline) is shown as a sensitivity analysis (**Supplementary Table 2**).

#### 3.4.3 False-discovery rate control

To address the multiplicity arising from univariate tests, the Benjamini–Hochberg procedure was applied as a sensitivity check (**Supplementary Table 3**). After FDR correction, five variables remained statistically significant (q < 0.05): WBC count, prior myocardial infarction, smoking, regional wall motion abnormalities, and eGFR. The primary variable-selection rule, however, was based on unadjusted p < 0.05 with forced inclusion of pre-specified clinical covariates, as pre-defined in the analysis plan; the FDR-adjusted results are presented for transparency.

#### 3.4.4 Sensitivity analysis excluding posterior wall MI

Given the borderline univariate significance of posterior wall MI (p = 0.050), Model A was fitted with posterior wall MI removed while retaining all other 14 covariates (**Supplementary Table 4**). The set of independent predictors and effect estimates remained largely consistent with the primary model, with an AUC of 0.871 (vs. 0.881 in the primary model). The likelihood-ratio test favoured the inclusion of posterior wall MI (LRT χ² = 6.31, df = 1, p = 0.012), supporting its retention in the final model. Overall, these sensitivity analyses confirmed the robustness of the primary findings.

### 3.5 In-hospital observation and HF severity

Total hospital length of stay was similar in the HF and non-HF groups (7.923 ± 1.229 vs. 7.934 ± 1.199 days; Welch t = -0.062, P = 0.951), indicating a comparable duration of in-hospital observation. Among the 65 patients who developed HF, the maximum Killip class was II in 27 patients (41.5%), III in 25 (38.5%), and IV in 13 (20.0%). The analytical dataset did not contain a reliable timestamp for the first HF episode after PCI; therefore, the exact PCI-to-HF interval could not be analyzed.(Additional PCI procedural characteristics are presented in **Supplementary Table 5**).

During hospitalization after emergency PCI, major cardiovascular complications were :cardiac death occurred in 1 patient (0.3%), cardiogenic shock in 4 patients (1.3%), malignant arrhythmias in 9 patients (2.9%), mechanical circulatory support was required in 1 patient (0.3%), and recurrent acute myocardial infarction occurred in 4 patients (1.3%). Compared with patients without in-hospital HF, patients who developed HF showed numerically higher rates of major cardiovascular complications, although statistical significance was not observed, likely due to the low event numbers.(**Supplementary Table 6** summarizes the incidence of major in-hospital cardiovascular complications according to the occurrence of HF).

## 4 Discussion

HF is a major adverse complication in patients with STEMI and may occur during the acute or subacute phase of STEMI (12). In recent years, although emergency PCI has significantly improved short-term survival in patients with STEMI, those with concomitant T2DM remain at high risk of developing in-hospital HF after undergoing emergency PCI (5).

As a major risk factor for coronary artery disease, T2DM is strongly associated with an increased risk of HF following STEMI. Previous studies have shown that, compared with non-diabetic patients, individuals with T2DM have an approximately 60%–70% higher risk of HF after STEMI (13). Even after adjustment for diabetes-related comorbidities, diabetes itself is associated with a 30%–42% increase in HF risk (14). The elevated cardiovascular risk in patients with T2DM is attributable not only to its role as a key risk factor for coronary artery disease but also to the clustering of multiple risk factors and suboptimal implementation of guideline-directed therapies (5). Therefore, identifying specific risk factors associated with HF in patients with T2DM complicated by STEMI following emergency PCI is of important clinical significance for improving short-term prognosis.

This single-center retrospective cohort study of 306 patients with T2DM and STEMI undergoing emergency PCI identified several independent associations with new-onset in-hospital HF. Positive associations were observed for prior myocardial infarction, smoking, regional wall motion abnormalities, higher admission WBC count, higher admission systolic blood pressure, and age ≥75 years. Hemoglobin and LVEF were inversely associated with HF, meaning that lower values corresponded to greater HF odds. Multiple sensitivity analyses, including restricted cubic spline age modeling and exclusion of borderline-significant posterior wall MI, provided some support for the robustness of the main associations. The model showed good internal discrimination and calibration, but the limited number of HF events relative to the number of estimated coefficients and the absence of external validation preclude immediate clinical implementation.

History of previous MI was the strongest independent predictor of post-PCI in-hospital HF in our cohort (OR = 6.205, P < 0.001). This result is consistent with established cardiovascular pathophysiological theories and previous clinical observations (15–17). Patients with prior MI have irreversible myocardial scarring, impaired myocardial reserve and pre-existing subclinical ventricular remodeling. When superimposed with new acute STEMI-related ischemic injury, the heart loses compensatory capacity to counteract pressure and volume overload, ultimately progressing to clinical heart failure despite timely revascularization (18, 19). This finding highlights the necessity of standardized long-term secondary prevention for patients with established coronary artery disease: strict blood glucose, lipid and blood pressure control, smoking cessation, and guideline-directed anti-heart failure medication should be implemented to reduce recurrent ischemic events and subsequent decompensation (20).

Smoking represented another powerful independent risk factor for in-hospital HF in T2DM-STEMI patients(OR = 5.552, P < 0.001). Beyond its well-known role in promoting atherosclerosis and acute myocardial infarction, tobacco exposure exerts multi-layered cardiac toxic effects specific to the acute infarction setting (21). Nicotine induces systemic vasoconstriction, increases myocardial oxygen consumption, exacerbates platelet aggregation and microthrombosis, while oxidative stress and inflammatory cascade further damage coronary microcirculation after PCI, amplifying reperfusion injury and expanding the infarct zone (22). In diabetic patients already burdened with baseline endothelial dysfunction, smoking synergistically accelerates maladaptive left ventricular remodeling, greatly raising the likelihood of acute pump failure during hospitalization (23). Our observation reinforces that inpatient smoking cessation intervention and long-term abstinence guidance are indispensable components of acute STEMI management for diabetic patients.

Baseline peripheral WBC count, a simple marker of systemic inflammation, was independently associated with higher HF risk in our study (OR = 1.143, P < 0.001), consistent with previous reports in diabetic acute coronary syndrome cohorts (24, 25). Myocardial necrosis triggered by STEMI activates robust inflammatory infiltration, and higher leukocyte levels reflect a more intense inflammatory response to ischemic injury. Overactivated inflammatory mediators aggravate microvascular no-reflow, expand myocardial edema, and drive early ventricular remodeling, collectively predisposing patients to acute heart failure (26–28). Notably, Benjamini–Hochberg FDR correction confirmed WBC count as one of the five most robust predictors of in-hospital HF, further supporting its clinical value as an inexpensive, rapid risk screening biomarker upon admission.

Lower admission hemoglobin was independently associated with greater odds of in-hospital HF. The OR of 0.971 was estimated per 1 g/L increase in hemoglobin; thus, each 1 g/L higher hemoglobin level corresponded to approximately 2.9% lower HF odds, and the clinically relevant interpretation is that lower hemoglobin is associated with greater HF odds. This direction is consistent with the negative regression coefficient. Reduced oxygen-carrying capacity during acute myocardial ischemia may exacerbate the imbalance between myocardial oxygen supply and demand, while lower hemoglobin may also reflect greater comorbidity burden, malnutrition, or chronic inflammation in patients with T2DM (29). Because no prespecified anemia threshold was analyzed, these findings should be interpreted as a continuous association rather than as evidence for a treatment threshold.

LVEF was also inversely associated with in-hospital HF. The OR of 0.953 was estimated per 1 percentage-point increase in LVEF, corresponding to approximately 4.7% lower HF odds for each percentage-point increase; equivalently, lower LVEF was associated with higher HF odds. LVEF reflects left ventricular systolic function, and lower values may indicate greater acute myocardial injury, impaired cardiac output reserve, and elevated filling pressure (30, 31). This finding supports the prognostic relevance of early echocardiographic assessment, while the modest effect per unit and the observational design warrant cautious interpretation.

Higher admission systolic blood pressure was independently associated with HF (OR = 1.018 per mmHg, P = 0.032), a finding that appears counterintuitive in acute STEMI and should not be interpreted as evidence that blood-pressure lowering would prevent HF. Admission SBP may partly reflect acute pain, sympathetic activation, catecholamine release, arterial stiffness, and other unmeasured markers of infarct severity or physiological stress (32–36). Reported J-shaped associations between admission SBP and adverse outcomes further suggest that both low and high pressures may identify vulnerable patients. Accordingly, the present association is best viewed as a risk-marker signal requiring confirmation rather than a causal treatment target.

Compared with patients younger than 60 years, patients aged ≥75 years exhibited a nearly 5-fold higher risk of in-hospital HF(OR = 4.792, P = 0.013). Advanced age is accompanied by age-related myocardial fibrosis, diastolic dysfunction, decreased physiological reserve, and accumulated multiple chronic comorbidities including long-standing T2DM, renal impairment and coronary multivessel disease (37). When confronted with the acute ischemic stress of STEMI, aged hearts lack sufficient compensatory capacity to maintain stable hemodynamics, leading to frequent acute HF episodes during hospitalization (38). Box–Tidwell linearity testing verified a non-linear relationship between age and HF risk, and categorical age stratification (<60, 60–74, ≥75 years) better captured this age-dependent risk gradient than linear age modeling or restricted cubic splines, with superior clinical interpretability for routine risk grouping.

Several variables with significant univariate associations lost statistical significance after full covariate adjustment, which warrants clinical discussion. First, estimated glomerular filtration rate (eGFR) showed significant between-group differences in baseline comparison and remained one of five FDR-significant univariate predictors, yet became non-significant in multivariable models. This suggests that renal dysfunction’s prognostic effect is largely mediated by overlapping risk factors including anemia, advanced age and chronic inflammation. Second, atrial fibrillation was significantly correlated with in-hospital HF in univariate analysis but failed to maintain independence after adjustment, possibly due to small event numbers of new-onset AF in our cohort. Third, procedural indicators including door-to-balloon time and chest pain-to-balloon delay showed only borderline univariate significance without independent predictive power, which may relate to the relatively narrow time window of reperfusion in our single-center emergency PCI system; stratified analysis of prolonged ischemia (>6 h) in larger cohorts is required to clarify its interaction with HF risk.

Notably, GLP-1 RA and SGLT2i use showed no between-group difference in the available dataset. The use of these agents was defined as pre-admission chronic medication use according to medication records obtained before hospitalization. However, medication exposure was recorded only as a binary use indicator, without detailed information on treatment duration, dosage, adherence, or changes in therapy before admission. Therefore, the present study cannot determine the relationship between the duration or intensity of chronic exposure to these agents and the risk of acute in-hospital HF, and the absence of a statistically significant association should not be interpreted as evidence of absence of cardiovascular benefit. Prospective studies incorporating detailed, time-resolved medication exposure, adherence information, and standardized treatment documentation are warranted to further clarify the potential effects of chronic GLP-1 RA and SGLT2i therapy on acute HF risk in this population.

Although patients who developed in-hospital HF demonstrated a higher clinical risk profile, the incidence of other severe cardiovascular complications remained relatively low. This may partly reflect timely emergency PCI management and the relatively short observation period confined to the index hospitalization. Longer-term follow-up studies are needed to determine whether in-hospital HF is associated with subsequent cardiovascular events beyond discharge.

### 4.1 Limitations

Several limitations should be acknowledged. First, this was a single-center retrospective observational study, limiting generalizability and precluding causal inference. Second, only 65 HF events were available for a model with 15 estimated coefficients, creating a meaningful risk of overfitting despite 10-fold cross-validation and bootstrap optimism correction; no *a priori* sample-size calculation was performed, and the model should be considered exploratory. Third, although total hospital length of stay was comparable between groups, the exact interval from PCI to first HF onset was unavailable, contrast volume could not be reliably retrieved for all patients from the retrospective procedural records and was therefore not included in the analysis, limiting the completeness of the available PCI-related procedural data. Fourth, information on diabetes duration, longitudinal or pre-admission glycemic control, medication adherence was limited. Furthermore, although baseline use of GLP-1 receptor agonists and SGLT2 inhibitors was assessed, we were unable to evaluate the effects of in-hospital initiation of these agents after STEMI. Future prospective studies are needed to determine whether early initiation of these therapies during hospitalization may influence acute heart failure outcomes. Fifth, the exclusion of chronic kidney disease referred to documented pre-existing disease; a single low admission eGFR was analyzed as renal dysfunction and may not reflect established chronic disease. Sixth, although multivariable regression revealed a negative association between posterior wall myocardial infarction and in-hospital HF, this finding was of only borderline significance in univariate analysis (P = 0.05) and lacks biological plausibility. We therefore attribute this observation to a statistical artifact or residual confounding bias likely driven by the limited sample size. This result warrants extremely cautious interpretation and should not be used to guide clinical decision-making at present. Finally, follow-up was limited to the index hospitalization, and no 30-day or long-term data on HF readmission, major adverse cardiovascular events, or mortality were available. Prospective multicenter studies with larger cohorts and independent external validation are required before clinical implementation of the model.

## 5 Conclusions

In this retrospective study of patients with T2DM and STEMI undergoing emergency PCI, prior myocardial infarction, smoking, regional wall motion abnormalities, higher WBC count, higher systolic blood pressure, and age ≥75 years were positively associated with in-hospital HF, whereas higher hemoglobin and LVEF were inversely associated with HF odds. The internally validated model demonstrated good discrimination and calibration, but the limited event number, retrospective single-center design, and lack of external validation mean that it should not yet be used for clinical decision-making. Independent prospective validation is required before assessing its role in individualized risk stratification.

## Data Availability

The original contributions presented in the study are included in the article/**Supplementary Material**. Further inquiries can be directed to the corresponding author.

## Supplementary material

Supplementary Table 1 Box–tidwell linearity check for continuous predictors in the multivariable model. β, regression coefficient of the X × ln(X) interaction; p, Wald p-value. The Box–Tidwell test assumes positive values; for variables with non-positive values an offset would be required. All variables in this model are positive.

Supplementary Table 2 Comparison of three approaches to modeling age. RCS, restricted cubic spline. Lower AIC/BIC indicates better fit. The original linear-age specification (B0) failed to detect the elevated HF risk in patients ≥ 75 (apparent OR = 1.01/year, p = 0.64). Both B1 and B2 reveal this effect; B2 is preferred for clinical interpretability while B1 confirms statistical non-linearity (joint LRT p < 0.001).

Supplementary Table 3 Benjamini–Hochberg FDR correction applied to all univariate tests. BH, Benjamini–Hochberg. q value = false-discovery-rate-adjusted p value. A q value < 0.05 means that, among all variables declared significant, the expected proportion of false discoveries is < 5%.

Supplementary Table 4 Sensitivity analysis — model A (without posterior wall MI). Model A statistics: AUC = 0.871 (vs. primary model AUC = 0.881); AIC = 237.78 (vs. 233.47); BIC = 293.64 (vs. 293.05). Bottom line: the substantive findings of the primary model are robust to removal of posterior wall MI; posterior wall MI is, however, statistically retained on the basis of the likelihood-ratio test.

Supplementary Table 5 Comparison of PCI procedural characteristics between HF and non-HF groups.

Supplementary Table 6 Incidence of major in-hospital cardiovascular complications according to heart failure status.
