# Supplementary material for: Factors associated with in-hospital heart failure after emergency percutaneous coronary intervention in patients with type 2 diabetes mellitus complicated by ST-segment elevation myocardial infarction: a retrospective study
Source: Front Endocrinol (Lausanne). 2026 Sep 3;17:1913869. doi: 10.3389/fendo.2026.1913869 (PMC13559705; doi:10.3389/fendo.2026.1913869)
Supplement: Supplementary file 1 [file SupplementaryFile1.docx]

**Table S1. Box–Tidwell Linearity Check for Continuous Predictors in the Multivariable Model**

| **Continuous predictor** | **Interaction β** | **Wald p** |
| --- | --- | --- |
| WBC count | -0.0856 | 0.4833 |
| eGFR | -0.0930 | 0.0945 |
| Hemoglobin | -0.0386 | 0.6684 |
| LVEF | +0.0008 | 0.9953 |
| Systolic BP | -0.1075 | 0.1573 |
| D-to-B time | +1.0612 | 0.1555 |
| Age | +0.5182 | 0.0055 |

*β, regression coefficient of the X × ln(X) interaction; p, Wald p-value. The Box–Tidwell test assumes positive values; for variables with non-positive values an offset would be required. All variables in this model are positive.*

**Table S2. Comparison of Three Approaches to Modeling Age**

| **Approach** | **AIC** | **BIC** | **10-fold CV AUC** | **Age effect (key)** |
| --- | --- | --- | --- | --- |
| B0: Age linear  (original) | 239.88 | 295.73 | 0.8311 | OR = 1.009/year, p = 0.641 (n.s.) |
| B1: Age RCS  (4 knots, sensitivity) | 224.66 | 287.96 | 0.8458 | Joint non-linearity LRT (2 df): χ² = 19.22,  p = < 0.001 |
| B2: Age categorical  (PRIMARY) | 233.47 | 293.05 | 0.8379 | 60–74 vs <60: OR = 0.84 (p = 0.67); ≥75 vs <60: OR = 4.79 (p = 0.01) |

*RCS, restricted cubic spline. Lower AIC/BIC indicates better fit. The original linear-age specification (B0) failed to detect the elevated HF risk in patients ≥ 75 (apparent OR = 1.01/year, p = 0.64). Both B1 and B2 reveal this effect; B2 is preferred for clinical interpretability while B1 confirms statistical non-linearity (joint LRT p < 0.001).*

**Table S3. Benjamini–Hochberg FDR Correction Applied to All Univariate Tests**

| **Variable** | **Unadjusted p** | **BH q value** |
| --- | --- | --- |
| WBC count (×10⁹/L) | < 0.001 | < 0.001 |
| Prior myocardial infarction | < 0.001 | < 0.001 |
| Smoking | < 0.001 | < 0.001 |
| Regional wall motion abnormalities | 0.002 | 0.016 |
| eGFR (mL/min/1.73 m²) | 0.005 | 0.039 |
| Hemoglobin (g/L) | 0.024 | 0.155 |
| Atrial fibrillation | 0.031 | 0.165 |
| LVEF (%) | 0.034 | 0.165 |
| Systolic BP (mmHg) | 0.048 | 0.192 |
| Posterior wall MI | 0.050 | 0.192 |
| Platelet count (×10⁹/L) | 0.059 | 0.192 |
| Time from chest pain to balloon (h) | 0.059 | 0.192 |
| Diabetes mellitus | 0.076 | 0.228 |
| Globulin (g/L) | 0.092 | 0.240 |
| D-to-B time (min) | 0.092 | 0.240 |
| HbA1c (%) | 0.099 | 0.242 |
| Serum potassium (mmol/L) | 0.119 | 0.273 |
| Hypertension | 0.131 | 0.278 |
| Occupation (employed vs. farmer) | 0.135 | 0.278 |
| Albumin (g/L) | 0.161 | 0.315 |

*BH, Benjamini–Hochberg. q value = false-discovery-rate-adjusted p value. A q value < 0.05 means that, among all variables declared significant, the expected proportion of false discoveries is < 5%.*

**Table S4. Sensitivity Analysis — Model A (Without Posterior Wall MI)**

| **Variable** | **β** | **OR** | **95% CI** | **p value** |
| --- | --- | --- | --- | --- |
| Prior myocardial infarction ★ | +1.671 | 5.316 | 2.365–11.953 | < 0.001 *** |
| Smoking ★ | +1.609 | 4.998 | 2.319–10.774 | < 0.001 *** |
| Regional wall motion abnormalities ★ | +0.894 | 2.444 | 1.124–5.314 | 0.024 * |
| WBC count (×10⁹/L) ★ | +0.137 | 1.147 | 1.077–1.222 | < 0.001 *** |
| eGFR (mL/min/1.73 m²) | -0.009 | 0.991 | 0.973–1.011 | 0.378 |
| Hemoglobin (g/L) ★ | -0.028 | 0.972 | 0.954–0.991 | 0.004 ** |
| Atrial fibrillation | +0.577 | 1.780 | 0.635–4.990 | 0.273 |
| LVEF (%) | -0.044 | 0.957 | 0.915–1.001 | 0.053 # |
| Systolic BP (mmHg) ★ | +0.019 | 1.019 | 1.003–1.036 | 0.020 * |
| Age 60–74 (vs. < 60, ref.) | -0.118 | 0.888 | 0.406–1.944 | 0.767 |
| Age ≥ 75 (vs. < 60, ref.) ★ | +1.312 | 3.714 | 1.146–12.034 | 0.029 * |
| Sex (male) | +0.232 | 1.261 | 0.547–2.910 | 0.586 |
| Hypertension | +0.397 | 1.488 | 0.709–3.121 | 0.293 |
| D-to-B time (min) | +0.023 | 1.024 | 0.943–1.111 | 0.578 |

*Model A statistics: AUC = 0.871 (vs. primary model AUC = 0.881); AIC = 237.78 (vs. 233.47); BIC = 293.64 (vs. 293.05). Bottom line: the substantive findings of the primary model are robust to removal of posterior wall MI; posterior wall MI is, however, statistically retained on the basis of the likelihood-ratio test.*

**Supplementary Table S5. Comparison of PCI Procedural Characteristics Between HF and Non-HF Groups**

| **Procedural characteristics** | **HF Group**  **(n = 65)** | **Non-HF Group**  **(n = 241)** | **Statistic** | **p value** |
| --- | --- | --- | --- | --- |
| PCI procedure completed, n (%) | 65 (100.0%) | 241 (100.0%) | — | — |
| Culprit vessel, n (%) |  |  | χ² = 1.857 | 0.603 |
| LAD | 30 (46.2%) | 91 (37.8%) |  |  |
| RCA | 25 (38.5%) | 99 (41.1%) |  |  |
| LCX | 7 (10.8%) | 36 (14.9%) |  |  |
| LM | 3 (4.6%) | 15 (6.2%) |  |  |
| Pre-procedural TIMI flow grade, n (%) |  |  | χ² = 1.067 | 0.785 |
| TIMI 0 | 35 (53.8%) | 116 (48.1%) |  |  |
| TIMI 1 | 15 (23.1%) | 58 (24.1%) |  |  |
| TIMI 2 | 8 (12.3%) | 41 (17.0%) |  |  |
| TIMI 3 | 7 (10.8%) | 26 (10.8%) |  |  |
| Post-procedural TIMI flow grade, n (%) |  |  | χ² = 3.903 | 0.272 |
| TIMI 0 | 1 (1.5%) | 1 (0.4%) |  |  |
| TIMI 1 | 2 (3.1%) | 3 (1.2%) |  |  |
| TIMI 2 | 6 (9.2%) | 12 (5.0%) |  |  |
| TIMI 3 | 56 (86.2%) | 225 (93.4%) |  |  |
| Number of implanted stents, n (%) |  |  | χ² = 0.820 | 0.664 |
| 1 | 39 (60.0%) | 159 (66.0%) |  |  |
| 2 | 19 (29.2%) | 61 (25.3%) |  |  |
| ≥3 | 7 (10.8%) | 21 (8.7%) |  |  |
| Number of implanted stents, mean ± SD | 1.51 ± 0.68 | 1.44 ± 0.65 | t ≈ 0.74 | 0.460 |
| Complete revascularization, n (%) | 38 (58.5%) | 154 (63.9%) | χ² = 0.436 | 0.509 |

**Supplementary Table S6. Incidence of Major In-hospital Cardiovascular Complications According to Heart Failure Status**

| **Major in-hospital cardiovascular complications** | **HF group**  **(n=65)** | **Non-HF group (n=241)** | **Total**  **(n=306)** | **p value** |
| --- | --- | --- | --- | --- |
| Cardiac death | 0 (0%) | 1 (0.4%) | 1 (0.3%) | 0.999 |
| Cardiogenic shock | 1 (1.5%) | 3 (1.2%) | 4 (1.3%) | 0.999 |
| Malignant arrhythmias | 3 (4.6%) | 6 (2.5%) | 9 (2.9%) | 0.421 |
| Mechanical circulatory support | 0 (0%) | 1 (0.4%) | 1 (0.3%) | 0.999 |
| Recurrent acute myocardial infarction | 1 (1.5%) | 3 (1.2%) | 4 (1.3%) | 0.999 |
| Any major cardiovascular complication | 5 (7.7%) | 12 (5.0%) | 17 (5.6%) | 0.531 |
